# Consumption of melatonin supplement improves cardiovascular disease risk factors and anthropometric indices in type 2 diabetes mellitus patients: a double-blind, randomized, placebo-controlled trial

**DOI:** 10.1186/s13063-021-05174-z

**Published:** 2021-03-25

**Authors:** Hadi Bazyar, Ahmad Zare Javid, Hossein Bavi Behbahani, Fardin Moradi, Bahman Moradi Poode, Parichehr Amiri

**Affiliations:** 1 Student Research Committee, Ahvaz Jundishapur University of Medical Sciences, Ahvaz, Iran; 2 Department of Nutrition, School of Allied Medical Sciences, Ahvaz Jundishapur University of Medical Sciences, Ahvaz, Iran; 3 Student Research Committee, Tabriz University of Medical Sciences, Tabriz, Iran

**Keywords:** Type 2 diabetes mellitus, Melatonin, Blood pressure, New anthropometric indices

## Abstract

**Background:**

Diabetes mellitus is a common chronic disease. Dyslipidemia and hypertension are two complications that may develop in diabetic patients if hyperglycemia, insulin resistance, and weight gain are not controlled. This study investigated the effects of melatonin supplementation on some cardiovascular disease risk factors and anthropometric indices in patients with type 2 diabetes mellitus (T2DM).

**Materials and methods:**

In this double-blind, randomized, placebo-controlled trial, 50 T2DM patients were randomly allocated to intervention and control groups which received two tablets of either melatonin or placebo (250 mg) once a day for 8 weeks. Systolic blood pressure (SBP), mean arterial pressure (MAP), pulse pressure (PP), the atherogenic index of plasma (AIP), weight, body mass index (BMI), waist and hip circumference (WC, HC), a body shape index (ABSI), abdominal volume index (AVI), body adiposity index (BAI), lipid accumulation product (LAP), conicity index, and waist-to-height ratio (WHtR) were evaluated in all the patients pre- and post-intervention.

**Results:**

Melatonin supplementation for 8 weeks significantly decreased the mean levels of SBP, MAP, PP, weight, BMI, WC, HC, BAI, AVI, conicity index, and WHtR post-intervention (*p* <  0.05). Also, the median changes of SBP, MAP, PP, weight, BMI, WC, HC BAI, AVI, and conicity index were significantly lower in the intervention group compared with the control group (*p* <  0.05). A significant increase (*p* <  0.001) was observed in the mean levels of ABSI in the intervention group. The median changes of ABSI were significantly greater in the intervention group compared with the control group (*p* <  0.001).

**Conclusions:**

Consumption of melatonin supplement may be effective in controlling arterial pressure including SBP, MAP, and PP and anthropometric indices (as predictors of obesity) in T2DM patients.

**Trial registration:**

Iranian Registry of Clinical Trials IRCT20190303042905N1. Registered on 17 May 2019.

## Background

There are clusters of metabolic abnormalities in type 2 diabetes mellitus (T2DM), including hyperglycemia, insulin resistance, and inflammation, which increase the risk of cardiovascular disease (CVD) [1]. CVD is one of the most important causes of mortality and morbidity among diabetic patients [2]. The prevalence rate of CVD in adults with diabetes is more than in adults without diabetes, and there is a strong association between increased fasting plasma glucose with a heightened risk of CVD [3]. On the other hand, T2DM patients are at a higher risk of developing dyslipidemia which is an important risk factor for coronary artery disease (CAD) [4]. Abnormal levels of serum lipids and lipoproteins may accelerate the progression of atherosclerosis in T2DM [3]. The atherogenic index of plasma (AIP) has recently been used as a better predictor of plasma atherogenicity [5]. AIP is defined as the logarithm [log] of the ratio of triglyceride (TG) levels to high-density lipoprotein (HDL) cholesterol. Studies indicate that there is a positive correlation between AIP and the risk of CVD [6].

Moreover, it was reported that T2DM and hypertension (HTN) may co-exist in some patients. The prevalence of HTN is approximately 30% in patients with T2DM [7]. The mean arterial pressure (MAP) reflects both peripheral resistance and cardiac output [8]. According to the “ADVANCE trial” [9], MAP was correlated with major CVD events, such that a 13% increase in risk was observed with a 13-mmHg increase in MAP. Pulse pressure (PP), the other indicator of CVD risk, is considered as a manifestation of arterial stiffness. Overall, in T2DM, dyslipidemia and HTN are considered as both quantitative and qualitative abnormalities including abnormal plasma levels of lipid profile and changes in the composition of lipoproteins [10], change that may lead to endothelial dysfunction and atherosclerosis [7, 11].

Melatonin is a neurohormone mostly secreted by the pineal gland and also locally by many other tissues during night [12]. In addition to the regulation of circadian rhythms, melatonin has other regulatory roles in inflammation, immune system, and oxidative stress [13–16]. Mukherjee et al. proposed that melatonin can provide cardio-protection at low pharmacological doses [17]. The ability of melatonin to improve cardiovascular function and its hypotensive effect because of its direct antioxidant and receptor-dependent actions suggest that melatonin may have some beneficial effects on controlling diabetic vascular complications [18, 19]. Moreover, animal studies pointed out that melatonin may reduce body weight and fat mass and regulate energy expenditure, glucose/lipid metabolism, and insulin secretion; therefore, it may play an effective role in weight management [20–22].

Obesity can be defined by different anthropometric measurements and indices. Recently, there has been growing speculation over which indices in obesity can calculate the CVD risk better [23]. Evidence from statistical analysis indicates that in order to detect CVD risk factors in populations, the measurements of centralized obesity (such as waist circumference (WC), waist-to-hip ratio (WHR), or waist-to-height ratio (WHtR)) are preferred to body mass index (BMI) [23–27]. Furthermore, many new anthropometric indices such as a body shape index (ABSI), abdominal volume index (AVI), body adiposity index (BAI), and lipid accumulation product (LAP) have been developed recently. These measures have been proven to be complementary indices to BMI and other known risk factors [28, 29]. In this study, we investigated the effects of melatonin supplementation on some CVD predictors such as MAP, PP, AIP, and anthropometric indices in T2DM patients.

## Material and method

### Participants

**Fig. 1:**
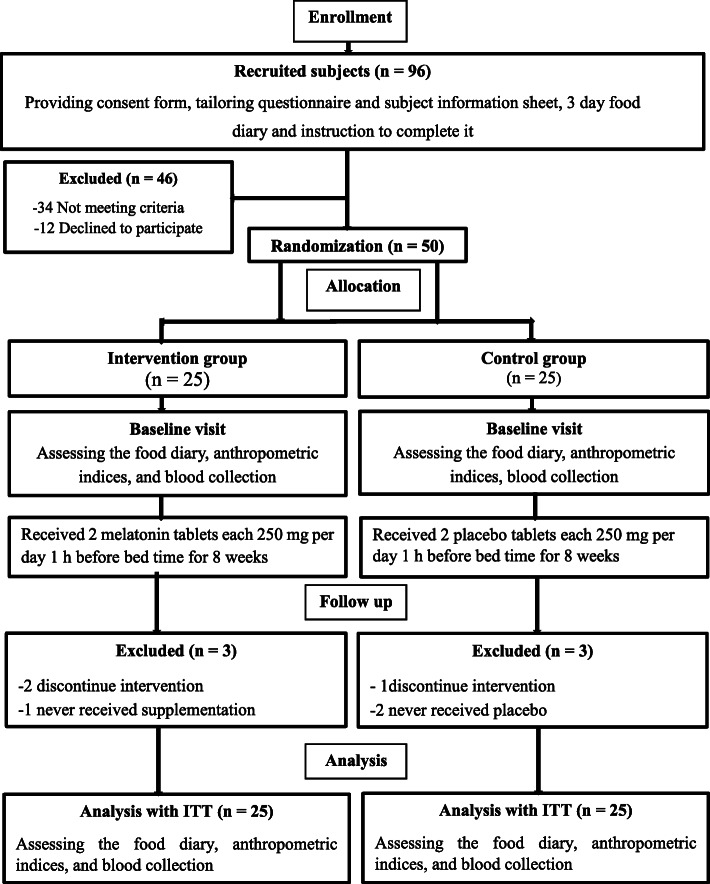
Stages of clinical trial progress

In this double-blinded, placebo-controlled trial, 96 T2DM patients were recruited from the endocrinology and metabolism clinics of Golestan Hospital of Ahvaz Jundishapur University of Medical Science, Iran. Fifty participants were selected based on the inclusion criteria (Fig. 1).

The FBS ≥ 126 mg/dl and HbA1c ≥ 6.5% or 2-hglucose (2 hpp) ≥ 200 mg/dl were used as the criteria to define diabetes mellitus (DM) [30]. The inclusion criteria were male or female, age of 30–60 years old, BMI 18.5–30 kg/m^2^, and having DM (for more than 5 years since the diagnosis). The exclusion criteria were having kidney and thyroid diseases; traveling for more than 2 weeks; pregnancy and lactation; smoking; having consumed alcohol over the past year; using medications such as immunosuppressive agents, insulin, anti-inflammatory drugs, and dietary supplements; and remarkable changes in using medications and treatment of diabetes or considerable changes in diet over the past 6 months. The compliance of the participants was assessed by counting the remaining tablets. Participants who consumed < 90% of the prescribed tablets were excluded.

The participants were allocated to intervention (*n* = 25) or placebo (*n* = 25) groups by a random block design. Accordingly, all the participants were assigned to six groups with four blocks in two steps plus two separate subjects using two codes of A and B according to the block design (AABB, BBAA, ABAB, BABA, ABBA, BABA). Assignment of patients in each of the study groups (supplement or placebo) was done by “Random allocation software” using classified randomized blocking method (block size, 4). In addition, in order to reduce selection bias error, allocation concealment was used. This was done by assigning unit codes (two codes A and B) to each patient’s tablets. In this way, 50 patients were randomly allocated to placebo (*n* = 25) or intervention (*n* = 25) groups. In fact, each patient received a can containing code A or B, and eventually 25 patients received cans containing code A and 25 patients received cans containing code B. Randomization was performed by an independent third person in the healthcare system who was not informed of the study. The researchers and patients were not informed of the allocation of participants to the groups (double-blinded). The participants in the intervention and control groups received 250 mg of either melatonin supplement (containing sodium starch glycolate, magnesium stearate, and 3 mg of net melatonin; purchased from the National Vitamin Company, Nature Made, USA) or placebo tablets (containing a few drops of peppermint oil, manufactured in the Faculty of Pharmacy, Ahvaz Jundishapur University of Medical Sciences, Iran). The melatonin and placebo tablets were prescribed for 8 weeks as two tablets once a day 1 h before bedtime. In terms of size, color, taste, and shape, the placebo and melatonin tablets were matched. The participants were asked to report any probable side effects of supplementation throughout the study. Patients were advised to follow any prescribed treatment and diabetic diet, and not to change their usual physical activity and diet pattern during the study.

### Blood pressure (BP), mean arterial pressure (MAP), and pulse pressure (PP)

BP was recorded in a relaxed sitting position on the left arm after at least a 5-min rest using a mercury sphygmomanometer. The measurement was repeated twice, and the mean of the two measurements was considered as the BP. MAP and PP were calculated according to the following equations [8, 9]:

$$ \mathrm{MAP}\left(\mathrm{mmHg}\right)=\left[\mathrm{SBP}+\left(2\times \mathrm{DBP}\right)\right]/3 $$

$$ \mathrm{PP}\left(\mathrm{mmHg}\right)=\mathrm{SBP}\left(\mathrm{mmHg}\right)-\mathrm{DBP}\left(\mathrm{mmHg}\right) $$

### Traditional and new anthropometric indices and nutritional assessment

All anthropometric measurements were performed by a trained nutritionist. Traditional anthropometric indices included height, weight, BMI, WC, and HC. Height (without shoes in a standard situation with a precision of 0.1 cm) and weight (by Seca scales, with light clothes, and a precision of 0.1 kg) were measured. BMI was calculated as weight (kg)/height (m^2^). WC as the smallest horizontal girth between the costal and iliac crests was measured using a non-stretchable measuring tape at minimal respiration. Hip circumference, as the greatest circumference at the level of greater trochanters (the widest portion of the hip) on both sides, was also measured.

All calculations for new anthropometric indices (ABSI, AVI, BAI, conicity, LAP, and WHtR) were performed according to the following equations [31]:

$$ \mathrm{A}\ \mathrm{body}\ \mathrm{shape}\ \mathrm{index}\ \left(\mathrm{ABSI}\right)=\frac{\mathrm{WC}}{{\mathrm{BMI}}^{2/3}\times {\mathrm{height}}^{1/2}} $$

$$ \mathrm{Abdominal}\ \mathrm{volume}\ \mathrm{index}\ \left(\mathrm{AVI}\right)=\left[2\Big({\mathrm{WC}}^2\right)+0.7\ \left(\mathrm{waist}/\mathrm{hip}\Big){}^2\right]/1000 $$

$$ \mathrm{Body}\ \mathrm{adiposity}\ \mathrm{index}\ \left(\mathrm{BAI}\right)=\frac{\mathrm{hip}}{{\mathrm{height}}^{1.5}}-18 $$

$$ \mathrm{Conicity}=\frac{\mathrm{WC}\left(\mathrm{m}\right)}{0.109\surd \frac{\mathrm{weight}\left(\mathrm{kg}\right)}{\mathrm{height}\left(\mathrm{m}\right)}} $$

$$ \mathrm{Lipid}\ \mathrm{accumulation}\ \mathrm{product}\ \left(\mathrm{LAP}\right)\ \mathrm{for}\ \mathrm{men}=\left[\mathrm{WC}\left(\mathrm{cm}\right)-65\right]\times \left[\mathrm{Triglyceride}\left(\mathrm{mM}\right)\right] $$

$$ \mathrm{LAP}\ \mathrm{for}\ \mathrm{women}=\left[\mathrm{WC}\left(\mathrm{cm}\right)-58\right]\times \left[\mathrm{Triglyceride}\left(\mathrm{mM}\right)\right] $$

Waist-to-height ratio (WHtR): WC (cm)/height (cm)

A 3-day 24-h dietary recall was also obtained for dietary assessment at the beginning and end of the study. The Nutritionist IV software (Axxya Systems, Stafford, TX, USA), modified for Iranian foods, was used for dietary data analysis.

### Atherogenic index of plasma (AIP)

AIP was obtained by dividing the logarithmically transformed ratio of triglycerides (TGs)/high-density lipoprotein cholesterol (HDL-C) ($\mathrm{AIP}=\log \frac{\mathrm{TG}}{\mathrm{HDL}}$) [5].

All the measurements and calculations were repeated after the intervention at the end of the study.

### Blood sampling and biochemical measurements

Blood samples of 2 cc were collected from all the participants after fasting overnight for at least 12 h and were centrifuged at 3000 rpm for 5 min. The lipid profile parameters were immediately measured using Pars Azmoon kits (Pars Azmoon, Tehran, Iran). The serum concentrations of TG and HDL-c were determined through an enzymatic procedure using glycerol phosphate oxidase and phosphotungstic acid, respectively.

### Statistical analysis

Statistical analyses were performed using SPSS software version 19.0 for Windows (PASW Statistics; SPSS Inc., Chicago, IL, USA). Analyses were conducted via an intention-to-treat (ITT) approach. The Kolmogorov–Smirnov test was used to check the normality of the data. An independent sample *t*-test and a paired sample *t*-test were also performed to compare the normal variables between and within groups, respectively, at baseline and after the intervention. Post-intervention, the analysis of covariance (ANCOVA) was done to compare the results between the two groups after adjustment for confounding factors. We used two models, a crude model (without adjustment) and with adjustment with age, sex, job, education, energy, physical activity, drugs, and disease duration (model 1). A Chi-square test was also used to compare the categorical variables. Data are expressed as mean ± standard deviation (SD) for normal distributions and median (Q1–Q3) for abnormal distributions. A Mann–Whitney *U* test was performed to compare changes between the two groups after the intervention. *P*-values < 0.05 were deemed significant.

Based on Ramezanpour et al.’s study [32], with 5% α (confidence interval of 95%) and 80% power of study, and considering SBP as the primary outcome, according to this formula: $n=\frac{{\left({z}_1-\frac{\alpha }{2}+{z}_1-\beta \right)}^2\left({\delta_1}^2+{\delta_2}^2\right)}{{\left({\mu}_1-{\mu}_2\right)}^2},$ the sample size was estimated to be 21 participants for each group; however, considering a 20% withdrawing rate, we selected 25 participants in each group.

## Results

Out of the 50 patients included in the study, six patients were excluded (three patients discontinued intervention, and three patients did not use the supplement or placebo). However, in the end, the final analysis was performed with the same 50 patients using the ITT method. The percentage of female participants was about 68.18% in the two groups (*p* = 0.51). The mean age of the participants was matched between the two groups (51.45 vs 53.71 years). There were no significant differences in terms of age and anthropometric indices between control and intervention groups at the baseline (Table 1). Also, dietary parameters including total energy, total fat, protein, and carbohydrate intakes did not differ between the two groups at the baseline (Table 2). There was not any report of adverse events or side effects.

**Table 1:** The characteristics of subjects at baseline

Variable	Control group (*n* = 25)	Intervention group (*n* = 25)	*p*-value*
Age (years)	51.52 ± 6.38	53.64 ± 4.82	0.19
Female/male (*N*)	18/ 7	16 / 9	0.54**
Disease duration (years)	7.36 ± 2.70	7.92 ± 2.48	0.44
Physical activity (met-min/week)	314.16 ± 165.44	303.64 ± 177.36	0.82
Weight (kg)	72.84 ± 6.70	73.88 ± 8.85	0.64
Height (m)	1.63 ± 0.08	1.64 ± 0.07	0.66
BMI (kg/m^2^)	27.42 ± 2.01	27.38 ± 2.16	0.94
WC (cm)	102.00 ± 8.45	101.60 ± 10.07	0.88
HC (cm)	107.80 ± 8.69	106.76 ± 10.04	0.69
FBG (mg/dl)	163.44 ± 33.48	172.04 ± 34.18	0.22
SBP (mmHg)	120.80 ± 16.30	124.00 ± 15.27	0.47
DBP (mmHg)	74.80 ± 9.62	74.00 ± 8.66	0.75
MAP (mmHg)	90.13 ± 10.56	90.66 ± 9.90	0.85
PP (mmHg)	46.00 ± 13.22	50.00 ± 11.54	0.26
HDL-c (mg/dl)	42.36 ± 11.10	43.88 ± 7.56	0.57
TG (mg/dl)	141.64 ± 41.47	161.12 ± 48.71	0.13
AIP	0.52 ± 0.18	0.55 ± 0.11	0.42
LAP (cm.mmol.l)	66.68 ± 23.61	77.74 ± 39.57	0.23
ABSI (m^11/6^ kg^−2/3^)	0.037 ± 0.009	0.037 ± 0.005	0.85
AVI	20.94 ± 3.38	20.84 ± 4.10	0.92
BAI (kg/m^2^)	32.88 ± 4.31	32.95 ± 5.68	0.96
Conicity	1.40 ± 0.10	1.42 ± 0.08	0.44
Waist-to-height ratio	0.62 ± 0.05	0.61 ± 0.05	0.66

Values are expressed as means ± SD. *p* < 0.05 was considered as significant

*p < 0.05 was considered as significant using the independent *T*-test between the two groups at baseline

**p < 0.05 was considered as significant using the Chi-square test

*BMI* body mass index, *WC* waist circumference, *HC* hip circumference, *FBG* fasting blood glucose, *SBP* systolic blood pressure, *DBP* diastolic blood pressure, *MAP* mean arterial pressure, *PP* pulse pressure, *TG* triglyceride, *HDL-c* high-density lipoprotein cholesterol, *AIP* atherogenic index of plasma, *ABSI* a body shape index, *AVI* abdominal volume index, *BAI* body adiposity index, *LAP* lipid accumulation product

**Table 2:** Mean ± SD of energy, carbohydrate, protein, fat intake at baseline and post-intervention

Variable		Baseline	post-intervention	P-value**
Energy (kcal/d)	Control group	1876.37 ± 158.34	1887.87 ± 176.99	0.97
	Intervention group	1924.83 ± 173.41	1875.74 ± 156.84	0.059
	P-value*	0.30	0.79	
Carbohydrate (g/d)	Control group	250.74 ± 22.62	248.65 ± 22.65	0.92
	Intervention group	244.64 ± 21.02	244.34 ± 20.32	0.44
	P-value*	0.32	0.48	
Protein (g/d)	Control group	76.83 ± 6.37	75.69 ± 6.30	0.91
	Intervention group	74.97 ± 6.39	74.86 ± 6.09	0.14
	P-value*	0.30	0.63	
Fat (g/d)	Control group	68.15 ± 5.69	67.18 ± 5.68	0.43
	Intervention group	66.71 ± 5.81	67.35 ± 5.87	0.16
	P-value*	0.38	0.91	

*P <0.05 was considered as significant using Independent T-test between the two groups post-intervention

**P <0.05 was considered as significant using Paired T-test

### The effects of melatonin on mean arterial pressure (MAP), pulse pressure (PP), atherogenic index of plasma (AIP), and systolic blood pressure (SBP)

Melatonin supplementation significantly decreased the mean levels of MAP (84.80 ± 8.33 vs 90.66 ± 9.90, respectively; *p* = 0.003), PP (50.00 ± 11.54 and 44.40 ± 11.21, respectively; *p* = 0.02), and SBP (114.40 ± 12.60 vs 124.00 ± 15.27, respectively; *p* = 0.004). The mean of MAP was significantly lower in the intervention group compared with the control group post-intervention (84.80 ± 8.33 vs 91.33 ± 8.81, respectively; *p =* 0.01). Also, this result remained significant after the adjustment of confounding factors (*p* = 0.01). The median (Q1–Q3) changes of MAP (− 3.33 (− 10.00, 0.00) vs 0.00 (− 3.33, 3.33), respectively; *p =* 0.005) were significantly lower in the intervention group in comparison with the control group. The mean of PP was not significantly different in the intervention group compared with the control group post-intervention (*p* > 0.05). But the median (Q1–Q3) changes of PP (0.00 (− 10.00, 0.00) vs 0.00 (− 10.00, 10.00), respectively; *p* = 0.03) were significantly lower in the intervention group in comparison with the control group. The mean of SBP was significantly lower in the intervention group compared with the control group post-intervention (114.40 ± 12.60 vs 122.80 ± 12.08, respectively; *p =* 0.02). The median (Q1–Q3) changes of SBP (0.00 (− 5.00, 10.00) vs 0.00 (− 10.00, 0.00), respectively; *p =* 0.003) were significantly lower in the intervention group in comparison with the control group.

No significant differences were observed in AIP within and between both groups (*p* ≥ 0.05) (Table 3).

**Table 3:** Atherogenic index of plasma, mean arterial pressure, pulse pressure, and old and new anthropometric indices at baseline and post-intervention

Variable	Control group (*n* = 25)			Intervention group (*n* = 25)			*p*-value*	*p*-value**	*p*-value***
	Baseline	After 8 weeks	*p*-value	Baseline	After 8 weeks	*p*-value			
Weight (kg)	72.84 ± 6.70	72.98 ± 6.78	0.75	73.88 ± 8.85	70.28 ± 7.57	0.001	0.19	0.15	
Changes	− 1.00 (− 1.00, 1.00)			− 3.00 (− 6.50, − 1.50)					< 0.001
BMI (kg/m^2^)	27.42 ± 2.01	27.47 ± 2.03	0.76	27.38 ± 2.16	26.11 ± 2.39	0.002	0.03	0.06	
Changes	− 0.30 (− 0.39, 0.42)			− 1.17 (− 2.40, − 0.51)					< 0.001
WC (cm)	102.00 ± 8.45	102.00 ± 8.18	1.00	101.60 ± 10.07	99.48 ± 9.54	0.01	0.32	0.26	
Changes	0.00 (− 1.00, 1.00)			− 1.00 (−3.00, 0.00)					0.003
HC (cm)	107.80 ± 8.69	107.10 ± 7.17	0.45	106.76 ± 10.04	103.04 ± 9.32	0.001	0.09	0.12	
Changes	0.00 (− 1.00, 1.00)			− 2.00 (− 5.00, − 1.00)					< 0.001
FBG (mg/dl)	163.44 ± 33.48	160.72 ± 51.37	0.76	172.04 ± 34.18	151.72 ± 51.27	0.08	0.53	0.59	
Changes	− 1.00 (− 28.50, 11.00)			− 4.00 (− 52.50, 14.00)					0.71
SBP (mmHg)	120.80 ± 16.30	122.80 ± 12.08	0.26	124.00 ± 15.27	114.40 ± 12.60	0.004	0.02	0.05	
Changes	0.00 (− 5.00, 10.00)			0.00 (− 10.00, 0.00)					0.003
MAP (mmHg)	90.13 ± 10.56	91.33 ± 8.81	0.39	90.66 ± 9.90	84.80 ± 8.33	0.003	0.01	0.01	
Changes	0.00 (− 3.33, 3.33)			− 3.33 (− 10.00, 0.00)					0.005
PP (mmHg)	46.00 ± 13.22	47.20 ± 12.08	0.47	50.00 ± 11.54	44.40 ± 11.21	0.02	0.40	0.60	
Changes	0.00 (− 10.00, 10.00)			0.00 (− 10.00, 0.00)					0.03
AIP	0.52 ± 0.18	0.53 ± 0.18	0.62	0.55 ± 0.11	0.49 ± 0.15	0.06	0.45	0.77	
Changes	0.01 (− 0.04, 0.08)			− 0.08 (− 0.15, 0.05)					0.10
LAP (cm mmol l)	66.68 ± 23.61	68.00 ± 26.42	0.64	77.74 ± 39.57	71.20 ± 32.26	0.22	0.70	0.47	
Changes	− 1.71 (− 8.28, 11.17)			− 3.06 (− 23.87, 14.34)					0.42
ABSI (m^11/6^ kg^−2/3^)	0.037 ± 0.009	0.036 ± 0.008	0.12	0.037 ± 0.005	0.04 ± 0.004	< 0.001	0.05	0.18	
Changes	0.000 (− 0.001, 0.001)			0.002 (0.001, 0.005)					< 0.001
AVI	20.94 ± 3.38	20.93 ± 3.28	0.96	20.84 ± 4.10	19.96 ± 3.85	0.01	0.34	0.29	
Changes	0.00 (− 0.42, 0.40)			− 0.45 (− 1.10, 0.00)					0.004
BAI (kg/m^2^)	32.88 ± 4.31	31.72 ± 3.59	0.07	32.95 ± 5.68	29.74 ± 4.56	< 0.001	0.09	0.09	
Changes	− 1.04 (− 3.33, 0.45)			− 3.38 (− 5.36, − 0.92)					0.04
Conicity	1.40 ± 0.10	1.39 ± 0.10	0.90	1.42 ± 0.08	1.39 ± 0.08	0.001	0.88	0.65	
Changes	0.00 (− 0.01, 0.01)			− 0.01 (− 0.04, 0.00)					0.003
Waist-to-height ratio	0.62 ± 0.05	0.62 ± 0.04	0.98	0.61 ± 0.05	0.60 ± 0.05	0.02	0.21	0.22	
Changes	0.000 (− 0.006, 0.006)			− 0.006 (− 0.018, 0.000)					0.01

Values are expressed as means ± SD for normal data and median (Q1–Q3) for abnormal data. *p* < 0.05 was considered as significant using the paired *T*-test

*p < 0.05 was considered as significant using the independent *T*-test between the two groups post-intervention (crude model)

**p < 0.05 was considered as significant using analysis of covariance (ANCOVA) between the two groups post-intervention after the adjustment for confounding factors (model 1)

***p < 0.05 was considered as significant changes using the Mann–Whitney *U* test between the two groups post-intervention

*BMI* body mass index, *WC* waist circumference, *HC* hip circumference, *FBG* fasting blood glucose, *SBP* systolic blood pressure, *MAP* mean arterial pressure, *PP* pulse pressure, *TG* triglyceride, *HDL-c* high-density lipoprotein cholesterol, *AIP* atherogenic index of plasma, *ABSI* a body shape index, *AVI* abdominal volume index, *BAI* body adiposity index, *LAP* lipid accumulation product

### The effects of melatonin on anthropometric indices

Weight (70.28 ± 7.57 vs 73.88 ± 8.85, respectively; *p* = 0.001), BMI (26.11 ± 2.39 vs 27.38 ± 2.16, respectively; *p* = 0.002), WC (99.48 ± 9.54 vs 101.60 ± 10.07, respectively; *p* = 0.01), HC (103.04 ± 9.32 vs 106.76 ± 10.04, respectively; *p* = 0.001), BAI (19.96 ± 3.85 vs 20.84 ± 4.10, respectively; *p* = 0.01), AVI (19.96 ± 3.85 vs 20.84 ± 4.10, respectively; *p* = 0.01), conicity (1.39 ± 0.08 vs 1.42 ± 0.08, respectively; *p* = 0.001), and WHtR (0.60 ± 0.05 vs 0.60 ± 0.05, respectively; *p* = 0.02) were significantly decreased post-intervention.

The mean of BMI was significantly lower in the intervention group compared with the control group post-intervention (26.11 ± 2.39 vs 27.47 ± 2.03, respectively; *p =* 0.03). Also, the results remained close significant after adjusting for confounding variables (*p* = 0.06).

The median (Q1–Q3) changes of weight (− 3.00 (− 6.50, − 1.50) vs − 1.00 (− 1.00, 1.00), respectively; *p* <  0.001), BMI (− 1.17 (− 2.40, − 0.51) vs − 0.30 (− 0.39, 0.42), respectively; *p* <  0.001), WC (− 1.00 (− 3.00, 0.00) vs 0.00 (− 1.00, 1.00), respectively; *p* = 0.003), HC (− 2.00 (− 5.00, − 1.00) vs 0.00 (− 1.00, 1.00), respectively; *p* <  0.001), AVI (− 0.45 (− 1.10, 0.00) vs 0.00 (− 0.42, 0.40), respectively; *p* = 0.004), BAI (− 3.38 (− 5.36, − 0.92) vs − 1.04 (− 3.33, 0.45), respectively; *p* = 0.04), conicity (− 0.01 (− 0.04, 0.00) vs 0.00 (− 0.01, 0.01), respectively; *p* = 0.003), and WHtR (− 0.006 (− 0.018, 0.000) vs 0.00 (− 0.006, 0.006), respectively; *p* = 0.01) were significantly lower in the intervention group in comparison with the control group (Table 3).

A significant increase was observed in the mean levels of ABSI in the intervention group (0.04 ± 0.004 and 0.037 ± 0.005, respectively; *p* < 0.001). The median (Q1–Q3) changes of ABSI were significantly greater in the intervention group compared with the control group (0.002 (0.001, 0.005) vs 0.000 (− 0.001, 0.001), respectively; *p* < 0.001) (Table 3).

## Discussion

This study was the first to investigate the effects of melatonin supplementation on predictors of CVDs in T2DM patients. In this study, AIP did not change. Although melatonin increased the HDL levels, the changes in AIP were not significant. There is evidence on the effect of melatonin on lipid profile and metabolism; however, such data are mainly reported from animal studies [33–35], and the results are controversial in humans [36–39]. Consistent with our study, Tamura et al. reported positive results regarding HDL post-consumption of 1 mg of melatonin; still, no improvement was observed in terms of total cholesterol and TG levels in pre- and postmenopausal women [38]. Also, Garfinkel et al. [37] reported no changes in TG levels in type 2 diabetic patients after receiving 2 mg of melatonin supplement. In another study, Rezvanfar et al. reported that HDL level was increased in response to melatonin, while no changes were observed in the other lipid profile parameters [39].

The results of this study showed that melatonin supplementation for 8 weeks significantly decreased MAP, SBP, and PP. In Możdżan et al.’s study conducted on diabetic patients with HTN, the hypotensive effect of melatonin on MAP and SBP was reported to be significant at two dosages of 3 and 5 mg [40]. In a meta-analysis study, Hadi et al. reported a significant decrease in SBP after the intervention of melatonin in five randomized clinical trials [41].

Previous studies proposed that melatonin can regulate BP through multiple mechanisms. First, melatonin can directly act as a free radical scavenger and provide appropriate concentrations of nitrogen oxide (NO), a strong vasodilator. Second, it can indirectly improve the function of the endothelium and reduce the activity of the adrenergic system. Furthermore, it is suggested that melatonin can provide hypotensive effects by stimulating melatonin receptors in peripheral vessels and the central nervous system [42].

In this study, melatonin consumption for 8 weeks significantly decreased weight, WC, HC, BMI, WHR, AVI, BAI, and conicity index post-intervention. AVI is considered as an important indicator to evaluate fat accumulation in the abdominal region [43]. In Ehrampoush et al.’s study, it was indicated that AVI had the greatest correlation with body fat percentage [31]. There is no need to consider body weight in the calculation of BAI and AVI [44]. Conicity is more associated with body fat in men compared to women [31]. In addition, melatonin supplementation significantly increased ABSI, which can adjust WC for height and weight, while it shows a strong correlation with mortality rates [28]. The study performed by Amstrup et al. on postmenopausal women indicated that 1 year of treatment with melatonin (1 or 3 mg per night) reduced fat mass [45]. Koziróg et al. in a research on patients with metabolic syndrome reported a significant reduction in BMI after consuming melatonin supplement (5 mg/day for 2 months) [46]. In Mesri Alamdari et al.’s study, the participants were supplemented with a daily dose of 6 mg of melatonin along with a low-calorie diet for 40 days. The results revealed that all the participants had significantly reduced weight, BMI, and waist and hip circumference [47]. Similarly, Szewczyk-Golec et al. administered a 30-day calorie-restricted diet combined with melatonin supplementation for obese individuals and reported significant weight loss without affecting BMI [48]. To the best of our knowledge, this study was the first to evaluate the effects of melatonin on new anthropometric indices. A majority of previous studies used weight and BMI as the outcomes of the study. However, our findings confirmed the effect of melatonin on weight control. As it is indicated that these indices are considered as more useful indices to predict the risk of CVD compared with the other anthropometric indices in prior studies [49, 50]. Hence, AVI, conicity, ABSI, BAI, and WHR along with BMI can be utilized as an investigative tool in the field to detect problems associated with T2DM and monitor the improvements.

There are several physiologic actions suggested for melatonin’s contribution to weight reduction [20]. Firstly, its effect on sleep quality and also the regulation of appetite can impact food intake [13, 45]. In addition, melatonin can impact both brown and white adipose tissue and play a controlling role in energy flow from the adipose tissue, so it can regulate the metabolic rate and energy balance [51, 52]. Also, some studies on aged subjects and shift workers indicated that low levels of melatonin can induce sleep disorder, insulin resistance, glucose intolerance, and metabolic circadian disorganization, characterizing a state of chronodisruption leading to obesity [20, 53].

There were some limitations to this study, including the short duration of the intervention. Moreover, several exclusion criteria may have limited the generalizability of the results. Therefore, further studies with longer interventions and a larger trial with some form of stratification are required to confirm the positive effects of melatonin supplementation on the management of diabetes.

## Conclusion

It is concluded that melatonin supplementation may be effective in controlling arterial blood pressure including SBP, MAP, and PP and improving the anthropometric indices in T2DM patients.

## Data Availability

All the data is contained in the manuscript.

## Abbreviations

TG: Triglyceride
HDL: High-density lipoprotein
LDL: Low-density lipoprotein cholesterol
MAP: Mean arterial pressure
PP: Pulse pressure
AIP: Atherogenic index of plasma
ABSI: A body shape index
AVI: Abdominal volume index
BAI: Body adiposity index
LAP: Lipid accumulation product
